# Prenatal folic acid supplement/dietary folate and cognitive development in 4-year-old offspring from the Japan Environment and Children’s Study

**DOI:** 10.1038/s41598-023-36484-8

**Published:** 2023-06-12

**Authors:** Hidekazu Nishigori, Toshie Nishigori, Taku Obara, Taeko Suzuki, Miyuki Mori, Karin Imaizumi, Tsuyoshi Murata, Hyo Kyozuka, Yuka Ogata, Akiko Sato, Kosei Shinoki, Seiji Yasumura, Mitsuaki Hosoya, Koichi Hashimoto, Keiya Fujimori, The Japan Environment and Children’s Study (JECS) Group, Michihiro Kamijima, Shin Yamazaki, Yukihiro Ohya, Reiko Kishi, Nobuo Yaegashi, Koichi Hashimoto, Chisato Mori, Shuichi Ito, Zentaro Yamagata, Hidekuni Inadera, Takeo Nakayama, Tomotaka Sofue, Masayuki Shima, Hiroshige Nakamura, Narufumi Suganuma, Koichi Kusuhara, Takahiko Katoh

**Affiliations:** 1 Department of Development and Environmental Medicine, Fukushima Medical Center for Children and Women, Fukushima Medical University Graduate School of Medicine, 1 Hikarigaoka, Fukushima City, Fukushima 960‑1295 Japan; 2 Fukushima Regional Center for the Japan Environmental and Children’s Study, Fukushima, Japan; 3 Department of Pediatrics, Fukushima Medical University School of Medicine, Fukushima, Japan; 4 Department of Pharmaceutical Sciences, Tohoku University Hospital, Sendai, Japan; 5 Department of Maternal Nursing and Midwifery, Fukushima Medical University School of Nursing, Fukushima, Japan; 6 Department of Maternal Nursing, Fukushima Medical University Graduate School of Nursing, Fukushima, Japan; 7 Department of Obstetrics and Gynecology, Fukushima Medical University School of Medicine, Fukushima, Japan; 8 Department of Public Health, Fukushima Medical University School of Medicine, Fukushima, Japan; 9 Nagoya City University, Nagoya, Japan; 10 National Institute for Environmental Studies, Tsukuba, Japan; 11 National Center for Child Health and Development, Tokyo, Japan; 12 Hokkaido University, Sapporo, Japan; 13 Tohoku University, Sendai, Japan; 14 Fukushima Medical University, Fukushima, Japan; 15 Chiba University, Chiba, Japan; 16 Yokohama City University, Yokohama, Japan; 17 University of Yamanashi, Chuo, Japan; 18 University of Toyama, Toyama, Japan; 19 Kyoto University, Kyoto, Japan; 20 Osaka University, Suita, Japan; 21 Hyogo Medical University, Nishinomiya, Japan; 22 Tottori University, Yonago, Japan; 23 Kochi University, Nankoku, Japan; 24 University of Occupational and Environmental Health, Kitakyushu, Japan; 25 Kumamoto University, Kumamoto, Japan

**Keywords:** Health care, Medical research

## Abstract

We evaluated the association between maternal prenatal folic acid supplement use/dietary folate intake and cognitive development in 4-year-old offspring (N = 3445) using data from the Japan Environment and Children’s Study. Cognitive development was evaluated using the Kyoto Scale of Psychological Development 2001. Multiple regression analysis revealed that offspring of mothers who started using folic acid supplements pre-conception had a significantly higher language-social developmental quotient (DQ) (partial regression coefficient 1.981, 95% confidence interval 0.091 to 3.872) than offspring of mothers who did not use such supplements at any time throughout their pregnancy (non-users). Offspring of mothers who started using folic acid supplements within 12 weeks of gestation had a significantly higher cognitive-adaptive (1.489, 0.312 to 2.667) and language-social (1.873, 0.586 to 3.159) DQ than offspring of non-users. Regarding daily dietary folate intake from preconception to early pregnancy, multiple regression analysis revealed that there was no significant association with any DQ area in the 200 to < 400 µg and the ≥ 400 µg groups compared with the < 200 µg group. Maternal prenatal folic acid supplementation starting within 12 weeks of gestation (but not adequate dietary folate intake from preconception to early pregnancy) is positively associated with cognitive development in 4-year-old offspring.

## Introduction

Folate is important for fetal neurodevelopment and an essential cofactor in DNA, RNA synthesis, and DNA methylation processes ^1,2^. Folate is also essential for the development of neural tubes in the first 4 weeks of pregnancy, and previous studies have established that supplementation with folic acid, which is the synthetic form of folate, in mothers reduces the risk of neural tube defects ^3–5^. Previous studies have also suggested that prenatal folic acid supplementation or adequate dietary folate intake may be beneficial for the cognitive development of pregnant women’s offspring ^1,2,6,7^.

In a study based on the Japan Environment and Children’s Study (JECS) database, adequate maternal dietary folate intake from preconception to early pregnancy was positively associated with verbal cognitive development in 2-years-old offspring ^8^. However, in the same study, maternal prenatal folic acid supplement use was not significantly associated with verbal or nonverbal cognitive development in 2-years-old offspring ^8^.

The association between maternal prenatal folic acid/folate intake and neurodevelopment of the offspring may change as the offspring grow ^1,2,7^. Therefore, continuous evaluation of offspring growth is necessary. In this study, we evaluated the association between maternal prenatal folic acid supplement use/dietary folate intake and cognitive development in 4-year-old offspring using the JECS database.

## Experimental methods

### Ethical approval

The JECS protocol has been previously published ^9,10^. The JECS protocol was reviewed and approved by the Ministry of the Environment Institutional Review Board on Epidemiological Studies (No. 100910001) and the Ethics Committees of all participating institutions. The JECS was conducted in accordance with the Helsinki Declaration and other national regulations and guidelines. Written informed consent was obtained from all participants. Informed consent was obtained from a parent or a legal guardian for participants below 20 years old.

From the JECS Main Study, we extracted data from the Sub-Cohort Study, which comprised 5% of the participating offspring who were randomly selected and met the eligibility criteria ^11^. Of 100,148 children in the JECS Main Study, children born after April 1, 2013, met the eligibility criteria. (1) all questionnaire and medical record data from offspring and their mothers collected from the first trimester to 6 months of age, (2) biospecimens (except umbilical cord blood) from children and their mothers collected in the first to second/third trimester and delivery were randomly selected for each Regional Centre at regular intervals. Of 10,302 selected offspring, 5017 participated. Face-to-face assessment of neuropsychiatric development, body measurement, pediatrician’s examination, blood/urine collection for clinical testing and chemical analysis, and home visits (ambient and indoor air measurement and dust collection) are conducted. Face-to-face assessment of neuropsychiatric development conducted by trained personnel via the Kyoto Scale of Psychological Development 2001 (KSPD) for 4-year-old offspring ^11^. The profiles of the participating mothers, fathers, and offspring did not substantially differ between the main and Sub-Cohort Studies^11^. For the present study, we used the jecs-ta-20210401 dataset, which was released in April 2021 and revised in February 2022. The dataset contains the cognitive developmental results of 4-year-old offspring in the form of KSPD scores. Multiple-birth offspring were excluded from the study because we wanted to focus on offspring from singleton pregnancies.

### Design and participants

The JECS is a nationwide, prospective, birth cohort study involving 100,000 mother–offspring pairs, started in 2011^9,10^. It is ongoing and is planned to continue until the offspring turn 18. Trained examiners evaluated the cognitive development of approximately 5000 offspring selected for the Sub-Cohort Study of the JECS ^11^. The dataset of 4-years-old offspring’s test results was provided to us in 2021.

We followed the same method as we did for our previous study in which we used the Kyoto Scale of Psychological Development 2001 (KSPD) on 2-year-old offspring ^8^. The main differences were that we used the KSPD data of 4-year-old offspring and evaluated sex differences.

### Exposure: maternal folic acid supplement use

The Ministry of Health, Labor and Welfare in Japan recommends the intake of 400 µg/day of supplementary folic acid for pregnant women and women intending to become pregnant ^12^. A face-to-face interview was conducted with pregnant women to assess their use of folic acid and other supplements ^13,14^. In this study, multivitamin supplements were not considered folic acid supplements, as we did not have data on the contents of each multivitamin supplement.

Participants were classified into four groups, based on the time of initiation of folic acid supplementation: (1) preconception users (started before conception), (2) early post-conception users (within 12 weeks of gestation), (3) late post-conception users (after 12 weeks of gestation), and (4) non-users (non-use of folic acid supplements before conception and during gestation).

### Exposure: maternal dietary folate intake

A semi-quantitative food frequency questionnaire (FFQ) was used to estimate participants’ dietary folate intake from foods ^13^. The FFQ comprises a list of foods with standard portion sizes commonly consumed in Japan ^15^. The validity of the FFQ for the estimation of dietary folate intake in Japan has previously been established ^15^. The FFQ consisted of 172 food and beverage items and nine frequency categories, ranging from almost nothing to seven or more times per day for food and 10 or more glasses per day for beverages. Thereafter, the intake of 53 nutrients was calculated.

The mother’s FFQ was administered during the first and second trimesters of gestation, at a median of 14.6 (interquartile range: 12.0–17.9) weeks of gestation. Participants reported their frequency of food consumption over the previous year.

The FFQ was not designed to estimate folic acid ^13,15^. The Ministry of Health, Labor and Welfare in Japan recommends an estimated average requirement for total dietary folate, for example, from natural food sources, as follows: an intake of ≥ 200 µg/day for adult women and ≥ 400 µg/day for pregnant women ^12^. Therefore, the study participants were classified into three groups, according to daily dietary folate intake (< 200 µg, 200 µg to < 400 µg, and ≥ 400 µg).

### Outcome: psychological development of 4-year-old offspring

The KSPD is a standardized developmental assessment tool for Japanese children, covering cognitive-adaptive and language-social areas ^16,17^. These areas correspond to nonverbal and verbal cognitive development, respectively. Scores are combined to form the developmental quotient (DQ; in days), which is calculated by dividing the developmental age (in days) by the chronological age (in days) and multiplying the quotient by 100. To ensure reliability of administration of the KSPD, the interviewers were trained and certified by the JECS. Administrative procedures and evaluations were strictly standardized to ensure inter-interviewer reliability.

### Statistical analysis and covariables

We compared the mothers’ characteristics and their offspring’s cognitive developmental data via analysis of variance (ANOVA) and Tukey’s range test. Sex differences were also examined. Multiple regression analyses were used to assess the association between maternal prenatal folic acid intake/dietary folate intake and offspring psychological development.

First, multiple regression analyses were adjusted for maternal age at delivery, maternal body mass index (kg/m^2^) before pregnancy, infertility treatment, unexpected pregnancies, parity, marital status, maternal highest level of education, paternal highest level of education, maternal smoking status during pregnancy, paternal smoking status during pregnancy, maternal alcohol consumption during pregnancy, annual household income (× 10^3^ yen/year) during pregnancy, pregnancy complications, obstetric labor complications, mode of delivery, maternal neuropsychiatric disorders, and a six-item maternal Kessler Psychological Distress Scale (K6) score ≥ 5 during pregnancy^18–20^. Adjustments were also made for the sex of the offspring (not for subgroup analysis), the offspring’s birth weight, gestational week of delivery, breastfeeding at 18 months postpartum, family structure, maternal job status after delivery, day care center attendance, multivitamin supplement use, iron preparation use, and trace element use. Dietary intake (measured with the FFQ) included energy content and nutrients, including amino acids, n − 3 unsaturated fatty acids, Fe, Ca, vitamin A, vitamin B_12_, and vitamin C. No multicollinearity was observed in the multiple regression analysis. For reference, the parity and number of the offspring’s siblings were confirmed to be multicollinear. The total energy, protein, and Zn contents were also confirmed to be multicollinear.

Second, multiple regression analyses were adjusted for variables selected using a stepwise method.

All analyses were significant at a 0.05 probability of significance and were performed using SAS statistical software, version 9.4 (SAS Institute Inc., Cary, NC, USA).

## Results

**Figure 1:**
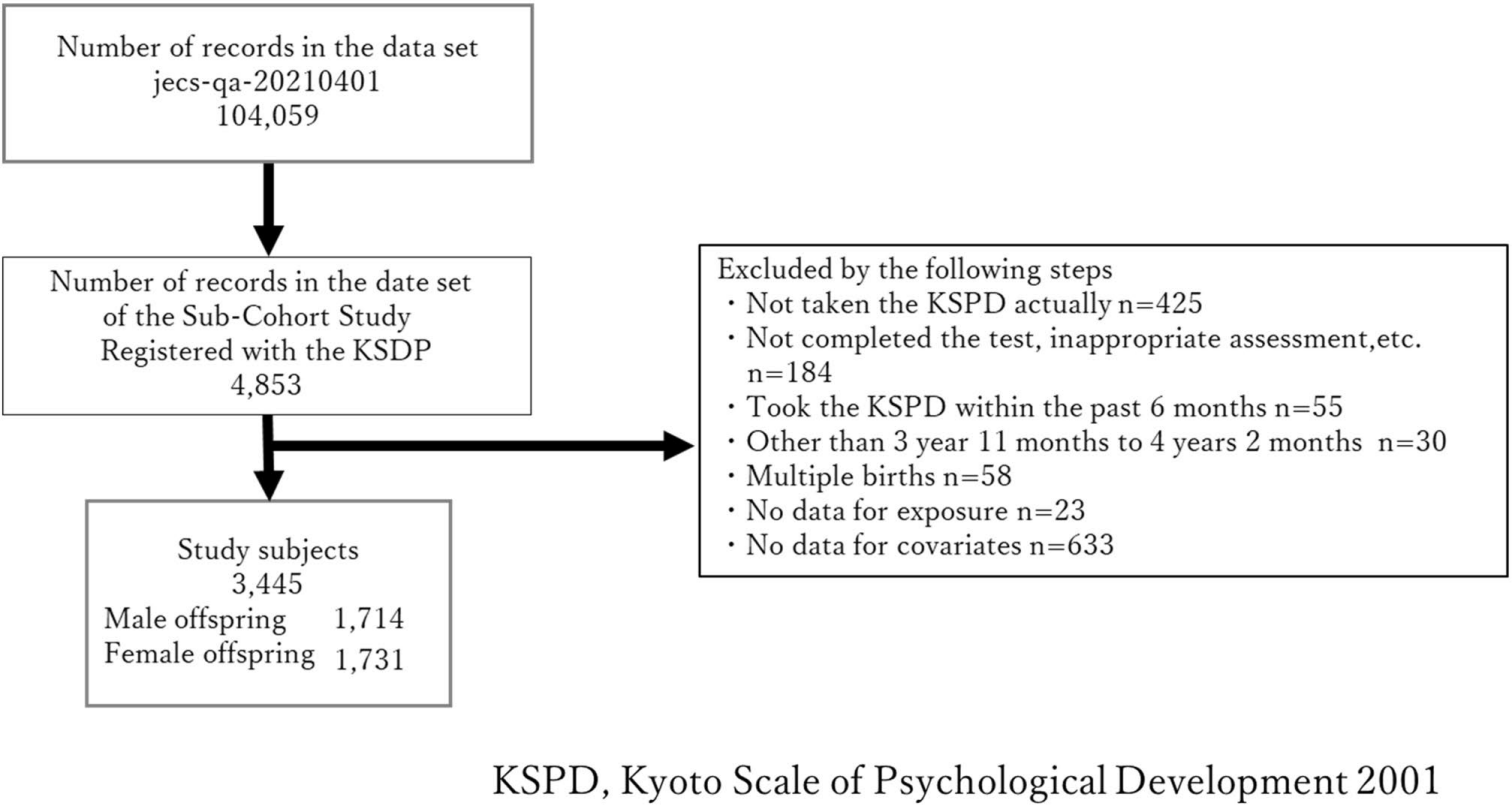
Participant selection process flow chart.

We analyzed the records of 3445 offspring out of the 104,059 records in the dataset (Fig. 1). Table 1 summarizes the participants’ characteristics. The maximum dietary folate intake in the ≥ 400 µg/day group was 2956 µg/day.

**Table 1:** Characteristics of the participants.

	Overall		Folic acid supplement user		Folic acid supplement non-user		Reference for multiple regression analysis
	(n = 3445)		(n = 1472)		(n = 1973)		
	n	%	n	%	n	%	
Maternal age at delivery							
Means +/− SD	32.1 + / − 4.8		32.4 +/− 4.7		31.8 + / − 4.9		Continuous variable
≤ 20	6	0.2	0	0.0	6	0.3	
20–24	194	5.6	55	3.7	139	7.1	
25–34	2104	61.1	909	61.8	1195	60.6	
≥ 35	1141	33.1	508	34.5	633	32.1	
Paternal age at delivery							
Means + / − SD	33.6 + / − 6.0		33.7 + / − 5.9		33.5 + / − 6.1		
≤ 20	1	0.0	0	0.0	1	0.1	
20–24	86	2.5	27	1.8	59	3.0	
25–34	1005	29.2	428	29.1	577	29.2	
≥ 35	795	23.1	340	23.1	455	23.1	
No answer	1558	45.2	677	46.0	881	44.7	
Maternal BMI (kg/m^2^) before pregnancy							
Means + / − SD	21.3 + / − 3.3		21.3 + / − 3.4		21.3 + / − 3.2		
< 18.5	545	15.8	228	15.5	317	16.1	
18.5 ≤–< 25.0	2543	73.8	1089	74.0	1454	73.7	Ref
≥ 25.0	357	10.4	155	10.5	202	10.2	
Infertility treatment							
No	3190	92.6	1325	90.0	1865	94.5	Ref
Yes	255	7.4	147	10.0	108	5.5	
Unexpected pregnancy							
No	3186	92.5	1396	94.8	1790	90.7	Ref
Yes	259	7.5	76	5.2	183	9.3	
Parity							
Primipara	1383	40.2	707	48.0	676	34.3	Ref
Multipara	2062	59.9	765	52.0	1297	65.7	
Marital status							
Married, common-law marriage	3409	99.0	1456	98.9	1953	99.0	Ref
Divorce	17	0.5	6	0.4	11	0.6	
Other	19	0.6	10	0.7	9	0.5	
Maternal highest level of education							
College, University	1566	45.5	738	50.1	828	42.0	
Senior high school	1785	51.8	697	47.4	1088	55.1	Ref
Junior high school	94	2.7	37	2.5	57	2.9	
Paternal highest level of education							
College, University	1482	43.0	705	47.9	777	39.4	
Senior high school	1790	52.0	708	48.1	1082	54.8	Ref
Junior high school	173	5.0	59	4.0	114	5.8	
Maternal smoking during pregnancy							
No	3348	97.2	1444	98.1	1904	96.5	Ref
Yes	97	2.8	28	1.9	69	3.5	
Paternal smoking during pregnancy							
No	2106	61.1	949	64.5	1157	58.6	Ref
Yes	1339	38.9	523	35.5	816	41.4	
Maternal alcohol consumption during pregnancy							
No	3030	88.0	1302	88.5	1728	87.6	Ref
Yes	415	12.1	170	11.6	245	12.4	
Annual household income (× 10^3^ yen/year) during pregnancy							
< 4000	1214	35.2	463	31.5	751	38.1	Ref
4000 ≤ –< 6000	1194	34.7	522	35.5	672	34.1	
≥ 6000	1037	30.1	487	33.1	550	27.9	
Pregnancy complications							
No	2870	83.3	1194	81.1	1676	85.0	Ref
Yes	575	16.7	278	18.9	297	15.1	
Obstetric labor complication							
No	1818	52.8	710	48.2	1108	56.2	Ref
Yes	1627	47.2	762	51.8	865	43.8	
Mode of delivery							
Vaginal	2850	82.7	1193	81.1	1657	84.0	Ref
Cesarean	595	17.3	279	19.0	316	16.0	
Maternal neuropsychiatric disorders							
No	3105	90.1	1316	89.4	1789	90.7	Ref
Yes	340	9.9	156	10.6	184	9.3	
Maternal Kessler 6 psychological distress scale score ≥ 5 during pregnancy							
No	2319	67.3	982	66.7	1337	67.8	Ref
Yes	1126	32.7	490	33.3	636	32.2	
Sex of offspring							
Male	1714	49.8	728	49.5	986	50.0	
Female	1731	50.3	744	50.5	987	50.0	
Birth weight of offspring(g)							
Means + / − SD	3050.6 + / − 401.7		3038.7 + / − 401.3		3059.4 + / − 401.9		Continuous variable
0 ≤–< 1500	3	0.1	1	0.1	2	0.1	
1500 ≤–< 2500	239	6.9	107	7.3	132	6.7	
2500 ≤–< 4000	3168	92.0	1351	91.8	1817	92.1	
≥ 4000	35	1.0	13	0.9	22	1.1	
Gestation week of delivery							
Means + / − SD	39.0 + / − 1.4		38.9 + / − 1.4		39.0 + / − 1.4		
22 ≤–< 28	1	0.0	0	0.0	1	0.1	
28 ≤–< 34	12	0.35	6	0.41	6	0.3	
34 ≤–< 37	128	3.7	64	4.4	64	3.2	
37 ≤–< 42	3299	95.8	1400	95.1	1899	96.3	Ref
≥ 42	5	0.2	2	0.1	3	0.2	
Breastfeeding at age of 18 months							
Yes	1240	32.3	534	33.0	706	31.8	
No	2599	67.7	1082	67.0	1517	68.2	Ref
Family structure							
Extended family	711	20.6	273	18.6	438	22.2	
Nuclear family	2734	79.4	1199	81.5	1535	77.8	Ref
Number of offspring’s siblings							
0	685	19.9	364	24.7	321	16.3	
1	1820	52.8	831	56.5	989	50.1	
≥ 2	940	27.3	277	18.8	663	33.6	
Maternal job after delivery							
No	1830	53.1	822	55.8	1008	51.1	Ref
Yes	1615	46.9	650	44.2	965	48.9	
Age at which the offspring started attending at daycare center							
Not attend	180	5.2	83	5.6	97	4.9	Ref
0 ≤–< 1	754	21.9	285	19.4	469	23.8	
1 ≤–< 2	762	22.1	327	22.2	435	22.1	
2 ≤–< 3	448	13.0	203	13.8	245	12.4	
≥ 3	1301	37.8	574	39.0	727	36.9	
FFQ: maternal dietary intake							
Gestational weeks of answer							
Median (IQR)	14.6 (12.0–17.9)		14.4 (12.0–17.6)		14.7 (12.0–18.3)		
Folate (μg/day)							
Median (IQR)	251 (187–340)		249 (188–339)		251 (187–341)		
0 ≤–< 200	1015	29.5	431	29.3	584	29.6	Ref
200 ≤–< 400	1896	55.0	822	55.8	1074	54.4	
400 ≤–< 1000	524	15.2	216	14.7	308	15.6	
≥ 1000 (maximum 2956)	10	0.3	3	0.2	7	0.4	
Total energy content (kJ/day)							
Median (IQR)	7108.6 (5828.3–8832.4)		7075.1 (5836.7–8663.0)		7108.6 (5828.3–8832.4)		Continuous variable
Protein (g/day)							
Median (IQR)	57.2 (45.6–73.2)		57.6 (45.9–73.1)		56.7 (45.2–73.3)		
Amino acids (g/day)							
Median (IQR)	21.5 (17.1–27.5)		21.8 (17.4–27.5)		21.4 (16.8–27.5)		Continuous variable
n − 3 unsaturated fatty acids (g/day)							
Median (IQR)	1.77 (1.29–2.35)		1.75 (1.31–2.35)		1.79 (1.27–2.36)		Continuous variable
Iron (mg/day)							
Median (IQR)	6.5 (5.2–8.4)		6.5 (5.2–8.4)		6.5 (5.1–8.4)		Continuous variable
Calcium (mg/day)							
Median (IQR)	453 (319–636)		461 (335–645)		448 (310–632)		Continuous variable
Zinc (mg/day)							
Median (IQR)	7.0 (5.7–8.8)		7.1 (5.7–8.8)		7.0 (5.7–8.9)		
Vitamin A (μgRAE/day)							
Median (IQR)	416 (281–634)		417 (286–625)		414 (276–639)		Continuous variable
Vitamin B12 (μg/day)							
Median (IQR)	3.9 (2.5–5.7)		3.9 (2.6–5.7)		3.9 (2.5–5.7)		Continuous variable
Vitamin C (mg/day)							
Median (IQR)	83 (55–122)		83 (55–120)		84 (56–124)		Continuous variable
Supplements or tablet							
Folic acid supplements							
No use	1973	57.3	0	0.0	1973	100.0	Ref
Preconception use	309	9.0	309	21.0	0	0.0	
Early post-conception use	890	25.8	890	60.46	0	0.0	
Late post-conception use	273	7.9	273	18.55	0	0.0	
Multivitamin supplements							
No	3317	96.3	1382	93.9	1935	98.1	Ref
Yes	128	3.7	90	6.1	38	1.9	
Iron preparations							
No	3392	98.5	1449	98.4	1943	98.5	Ref
Yes	53	1.5	23	1.6	30	1.5	
Trace element							
No	3224	93.6	1262	85.7	1962	99.4	Ref
Yes	221	6.4	210	14.3	11	0.6	
Kyoto scale of psychological development							
Cognitive-adaptive DQ							
Means + / − SD	96.3 + / − 14.2		97.0 + / − 14.5		95.8 + / − 13.9		
Language-social DQ							
Means + / − SD	96.0 + / − 15.8		97.7 + / − 15.9		94.7 + / − 15.5		

Folic acid supplement user : Included (1) preconception users (started before conception), (2) early post-conception users (within 12 weeks of gestation), (3) late post-conception users (after 12 weeks of gestation) Folic acid supplement non-user :non-use of folic acid supplements before conception and during gestation.

Abbreviations: body mass index (BMI), interquartile range (IQR), food frequency questionnaire (FFQ), Retinol activity equivalent (RAE), developmental quotient (DQ), standard deviation (SD).

The 6-item Kessler Psychological Distress Scale (K6; total point scores ranged from 0 to 24).

*IQR* interquartile range, *DQ* developmental quotient.

### Folic acid supplements

The results of the ANOVA and Tukey’s range test for maternal folic acid supplement use and the KSPD score of the offspring are summarized in Table 2.

**Table 2:** ANOVA for maternal folic acid supplement use and the Kyoto Scale of Psychological Development 2001 of 4-year-old offspring. (Mean values and standard deviations).

Folic acid supplements	n	%	Mean		SD	p	Tukey’s test
Overall (n = 3445)							
Cognitive-adaptive DQ							
No use	1973	57.27	95.8	+/−	13.9	0.03	
Preconception use	309	8.97	96.8	+ / −	14.2		
Early post-conception use	890	25.83	97.5	+ / −	14.3		*** (4)–(2)
Late post-conception use	273	7.92	95.8	+ / −	15.3		
Language-social DQ							
No use	1973	57.27	94.7	+ / −	15.5	< .0001	
Preconception use	309	8.97	98.7	+ / −	16.2		*** (4)–(1)
Early post-conception use	890	25.83	97.8	+ / −	15.5		*** (4)–(2)
Late post-conception use	273	7.92	96.6	+ / −	17.1		
Male offspring (n = 1714)							
Cognitive-adaptive DQ							
No use	986	57.53	94.8	+ / −	14.7	0.57	n.s
Preconception use	174	10.15	96.0	+ / −	13.7		
Early post-conception use	418	24.39	95.7	+ / −	15.0		
Late post-conception use	136	7.93	94.5	+ / −	16.1		
Language-social DQ							
No use	986	57.53	93.5	+ / −	16.1	< .0001	
Preconception use	174	10.15	98.6	+ / −	16.7		*** (4)–(1)
Early post-conception use	418	24.39	96.8	+ / −	16.2		*** (4)–(2)
Late post-conception use	136	7.93	94.2	+ / −	17.1		
Female offspring (n = 1731)							
Cognitive-adaptive DQ							
No use	987	57.02	96.8	+ / −	13.0	0.03	
Preconception use	135	7.8	97.8	+ / −	14.9		
Early post-conception use	472	27.27	99.0	+ / −	13.5		*** (4)–(2)
Late post-conception use	137	7.91	97.1	+ / −	14.5		
Language-social DQ							
No use	987	57.02	95.9	+ / −	14.7	0.002	
Preconception use	135	7.8	98.9	+ / −	15.6		
Early post-conception use	472	27.27	98.6	+ / −	14.7		*** (4)–(2)
Late post-conception use	137	7.91	99.0	+ / −	16.8		

Participants were classified into four groups based on folic acid supplementation start time: (1) preconception users (started before conception), (2) early post-conception users (within 12 weeks of gestation), (3) late post-conception users (after 12 weeks of gestation) and (4) non-users (non-use of folic acid supplements before conception and during gestation).

*** (4)–(1), *** (4)–(2), Results of Tukey’s range test indicate a significant difference at the 0.05 level of significance.

*DQ* evelopmental quotient, *SD* standard deviation, *n.s.* no significant difference.

Overall, the multiple regression analysis without the stepwise method revealed significantly higher scores for cognitive-adaptive DQ among early post-conception users (partial regression coefficient [B]: 1.489, 95% confidence interval [CI] 0.312 to 2.667, standardized partial regression coefficient [β]: 0.046, *P* = 0.01) than among non-users (Table 3). There was also a significantly higher score for language-social DQ among the preconception (B: 1.981, 95% CI 0.091 to 3.872, β: 0.036, *P* = 0.04) and early post-conception (B: 1.873, 95% CI 0.586 to 3.159, β: 0.052, *P* = 0.004) users than among non-users (Table 3). The multiple regression analysis with the stepwise method revealed significantly higher scores for cognitive-adaptive DQ among early post-conception users (B: 1.595, 95% CI 0.430 to 2.760, β: 0.049, *P* = 0.01) than among non-users (Table 3). There was also a significantly higher score for language-social DQ among the preconception (B: 2.122, 95% CI 0.273 to 3.972, β: 0.039, *P* = 0.02) and early post-conception (B: 1.932, 95% CI 0.718 to 3.145, β: 0.054, *P* = 0.002) users than among non-users (Table 3).

**Table3:** Multiple regression analysis for maternal folic acid supplement use and the Kyoto scale of psychological development 2001 of 4-year-old offspring. (coefficient values and 95% confidence intervals).

Folic acid supplements use	Bivariate analysis						Multiple regression analysis						Multiple regression analysis					
							Adjusted *1 and dietary folate intake						Adjusted *2 and dietary folate intake					
	R^2^	B	95%CI		β	p	R^2^	B	95%CI		β	p	R^2^	B	95%CI		β	p
Overall																		
Cognitive-adaptive DQ																		
No use	0.003	Ref					0.060	Ref					0.057	Ref				
Preconception use		0.963	− 0.738	2.663	0.019	0.27		0.387	− 1.343	2.116	0.008	0.66		0.642	− 1.059	2.344	0.013	0.46
Early post-conception use		1.671	0.549	2.793	0.052	0.004		1.489	0.312	2.667	0.046	0.01		1.595	0.430	2.760	0.049	0.01
Late post-conception use		− 0.024	− 1.819	1.770	0.000	0.98		− 0.061	− 1.837	1.716	− 0.001	0.95		0.012	− 1.754	1.778	0.000	0.99
Language-social DQ																		
No use	0.010	Ref					0.089	Ref					0.087	Ref				
Preconception use		4.036	2.155	5.917	0.073	< .0001		1.981	0.091	3.872	0.036	0.04		2.122	0.273	3.972	0.039	0.02
Early post-conception use		3.066	1.825	4.308	0.085	< .0001		1.873	0.586	3.159	0.052	0.004		1.932	0.718	3.145	0.054	0.002
Late post-conception use		1.898	− 0.087	3.883	0.033	0.06		1.116	− 0.826	3.058	0.019	0.26		1.052	− 0.880	2.983	0.018	0.29
Male offspring																		
Cognitive-adaptive DQ																		
No use	0.001	Ref					0.063	Ref					0.051	Ref				
Preconception use		1.193	− 1.194	3.579	0.024	0.33		0.693	− 1.775	3.160	0.014	0.58		0.758	− 1.630	3.146	0.015	0.53
Early post-conception use		0.932	− 0.762	2.626	0.027	0.28		1.223	− 0.574	3.019	0.036	0.18		1.104	− 0.664	2.872	0.032	0.22
Late post-conception use		− 0.315	− 2.969	2.340	− 0.006	0.82		0.205	− 2.474	2.884	0.004	0.88		− 0.333	− 2.961	2.294	− 0.006	0.80
Language-social DQ																		
No use	0.013	Ref					0.079	Ref					0.067	Ref				
Preconception use		5.114	2.487	7.741	0.094	0.0001		3.316	0.606	6.026	0.061	0.02		3.666	1.057	6.275	0.068	0.01
Early post-conception use		3.301	1.436	5.166	0.087	0.001		2.377	0.405	4.350	0.062	0.02		2.646	0.807	4.485	0.069	0.005
Late post-conception use		0.685	− 2.237	3.608	0.011	0.65		0.748	− 2.194	3.690	0.012	0.62		0.560	− 2.317	3.438	0.009	0.70
Female offspring																		
Cognitive-adaptive DQ																		
No use	0.005	Ref					0.066	Ref					0.056	Ref				
Preconception use		0.955	− 1.464	3.374	0.019	0.44		− 0.087	− 2.563	2.389	− 0.002	0.95		0.148	− 2.246	2.541	0.003	0.90
Early post-conception use		2.212	0.737	3.687	0.073	0.003		1.670	0.104	3.236	0.055	0.04		1.673	0.211	3.135	0.055	0.02
Late post-conception use		0.258	− 2.145	2.661	0.005	0.83		− 0.469	− 2.876	1.939	− 0.009	0.70		− 0.098	− 2.467	2.271	− 0.002	0.94
Language-social DQ																		
No use	0.009	Ref					0.113	Ref					0.105	Ref				
Preconception use		2.998	0.304	5.692	0.054	0.03		0.553	− 2.140	3.245	0.010	0.69		0.648	− 1.979	3.275	0.012	0.63
Early post-conception use		2.721	1.079	4.364	0.081	0.001		1.275	− 0.428	2.979	0.038	0.14		1.352	− 0.248	2.952	0.040	0.10
Late post-conception use		3.094	0.418	5.771	0.056	0.02		1.355	− 1.264	3.973	0.024	0.31		1.473	− 1.117	4.062	0.026	0.26

Participants were classified into four groups based on folic acid supplementation start time: (1) preconception use (started before conception), (2) early post-conception use (within 12 weeks of gestation), (3) late post-conception user (after 12 weeks of gestation) and (4) non-users (non-use of folic acid supplements before conception and during gestation).

*DQ* developmental quotient, *B* partial regression coefficient, *CI* confidence interval, *Β(beta)* standardized partial regression coefficients, *R2* coefficient of determination.

*1: Adjusted for maternal age at delivery, maternal body mass index (kg/m2) before pregnancy, infertility treatment, unexpected pregnancies, parity, marital status, maternal highest level of education, paternal highest level of education, maternal smoking status during pregnancy, and paternal smoking status during pregnancy, maternal alcohol consumption during pregnancy, annual household income during pregnancy, pregnancy complications, obstetric labor complications, mode of delivery, maternal neuropsychiatric disorders, maternal Kessler 6 (K6) psychological distress scale scores >  = 5 during pregnancy, offspring’s sex (for overall) and birth weight, gestation week of delivery, breastfeeding at age of 18 months, family structure, maternal job after delivery, day care center attendance, multivitamin supplement use, iron preparations, trace element use, and the dietary intake (FFQ) included energy content and nutrients, including amino acids, n-3 unsaturated fatty acids, iron, calcium, vitamin A, vitamin B12, vitamin C.

*2: Variable selection was performed using a stepwise method. Adjusted for “dietary folate intake”.

*2: Cognitive-adaptive DQ of Overall; Adjusted for maternal body mass index (kg/m2) before pregnancy, unexpected pregnancies, parity, marital status, maternal highest level of education, paternal highest level of education, paternal smoking status during pregnancy, maternal neuropsychiatric disorders, maternal Kessler 6 (K6) psychological distress scale scores >  = 5 during pregnancy, offspring’s sex (for overall) and birth weight, family structure, maternal job after delivery, day care center attendance, iron preparations, trace element use, and the dietary intake (FFQ) included vitamin A.

*2: Language-social DQ of Overall; Adjusted for maternal age at delivery, maternal body mass index (kg/m2) before pregnancy, unexpected pregnancies, parity, maternal highest level of education, paternal highest level of education, paternal smoking status during pregnancy, annual household income during pregnancy, pregnancy complications, maternal Kessler 6 (K6) psychological distress scale scores >  = 5 during pregnancy, offspring’s sex (for overall) and birth weight, maternal job after delivery, day care center attendance, iron preparations, and the dietary intake (FFQ) included calcium, vitamin B12.

*2: Cognitive-adaptive DQ of Male offspring; Adjusted for unexpected pregnancies, parity, marital status, maternal highest level of education, paternal highest level of education, paternal smoking status during pregnancy, offspring’s birth weight, day care center attendance, trace element use, and the dietary intake (FFQ) included vitamin B12.

*2: Language-social DQ of Male offspring; Adjusted for maternal age at delivery, maternal body mass index (kg/m2) before pregnancy, parity, maternal highest level of education, paternal highest level of education, paternal smoking status during pregnancy, maternal Kessler 6 (K6) psychological distress scale scores >  = 5 during pregnancy, offspring’s birth weight, iron preparations, and the dietary intake (FFQ) included vitamin B12.

*2: Cognitive-adaptive DQ of Female offspring; Adjusted for maternal highest level of education, paternal highest level of education, paternal smoking status during pregnancy, mode of delivery, maternal neuropsychiatric disorders, maternal Kessler 6 (K6) psychological distress scale scores >  = 5 during pregnancy, offspring’s birth weight, gestation week of delivery, family structure, day care center attendance.

*2: Language-social DQ of Female offspring; Adjusted for maternal age at delivery, unexpected pregnancies, parity, maternal highest level of education, paternal highest level of education, pregnancy complications, maternal neuropsychiatric disorders, family structure, maternal job after delivery, day care center attendance, iron preparations.

In male offspring, the multiple regression analysis without the stepwise method revealed significantly higher score for language-social DQ was observed among preconception (B: 3.316, 95% CI 0.606 to 6.026, β: 0.061, *P* = 0.02) and early post-conception (B: 2.377, 95% CI 0.405 to 4.350, β: 0.062, *P* = 0.02) users than among non-users (Table 3). The multiple regression analysis with the stepwise method that revealed significantly higher score for language-social DQ was observed among preconception (B: 3.666, 95% CI 1.057 to 6.275, β: 0.068, *P* = 0.01) and early post-conception (B: 2.646, 95% CI 0.807 to 4.485, β: 0.069, *P* = 0.005) users than among non-users (Table 3).

In female offspring, the multiple regression analysis without the stepwise method revealed significantly higher score for cognitive-adaptive DQ was observed among early post-conception users (B: 1.670, 95% CI 0.104 to 3.236, β: 0.055, *P* = 0.04) than among non-users (Table 3). The multiple regression analysis with the stepwise method that revealed significantly higher score for cognitive-adaptive DQ was observed among early post-conception users (B: 1.673, 95% CI 0.211 to 3.135, β: 0.055, *P* = 0.02) than among non-users (Table 3).

### Dietary folate intake

The results of the ANOVA and Tukey’s range test for maternal dietary folate intake and the KSPD score of offspring are summarized in Table 4.

**Table 4:** ANOVA for maternal folate intake from food and the Kyoto scale of psychological development 2001 of 4-year-old offspring. (mean values and standard deviations).

Folate (μg) diet per day	n	%	Mean		SD	p	Tukey’s test
Overall							
Cognitive-adaptive DQ							
0 ≤ – < 200	1015	29.46	95.8	+/−	14.1	0.30	n.s
200 ≤ – < 400	1896	55.04	96.7	+/−	14.1		
≥ 400	534	15.5	96.0	+/−	14.9		
Language-social DQ							
0 ≤ – < 200	1015	29.46	95.6	+/−	15.4	0.57	n.s
200 ≤ – < 400	1896	55.04	96.2	+/−	15.8		
≥ 400	534	15.5	95.9	+/−	16.3		
Male offspring							
Cognitive-adaptive DQ							
0 ≤ – < 200	497	29	94.5	+/−	14.5	0.05	n.s
200 ≤ – < 400	958	55.89	95.9	+/−	14.6		
≥ 400	259	15.11	93.6	+/−	16.1		
Language-social DQ							
0 ≤ – < 200	497	29	94.3	+/−	16.2	0.28	n.s
200 ≤ – < 400	958	55.89	95.4	+/−	16.1		
≥ 400	259	15.11	93.9	+/−	17.8		
Female offspring							
Cognitive-adaptive DQ							
0 ≤ – < 200	518	29.92	97.1	+/−	13.5	0.53	n.s
200 ≤ – < 400	938	54.19	97.5	+/−	13.5		
≥ 400	275	15.89	98.3	+/−	13.3		
Language-social DQ							
0 ≤ – < 200	518	29.92	96.9	+/−	14.5	0.75	n.s
200 ≤ – < 400	938	54.19	97.1	+/−	15.4		
≥ 400	275	15.89	97.7	+/−	14.6		

Results of Tukey's range test indicate a significant difference at the 0.05 level of significance.

*DQ* evelopmental quotient, *SD* standard deviation, *n.s.* No significant difference.

Overall, the multiple regression analysis without the stepwise method revealed no significant association with any DQ score in the 200 µg to < 400 µg group or the ≥ 400 µg group compared with the < 200 µg group (Table 5). The multiple regression analysis with the stepwise method revealed no significant association with any DQ score in the 200 µg to < 400 µg group or the ≥ 400 µg group compared with the < 200 µg group (Table 5).

**Table 5:** Multiple regression analysis for maternal folate intake from food and the Kyoto scale of psychological development 2001 of 4-year-old offspring. (coefficient values and 95% confidence intervals).

Folate (μg) diet per day	Bivariate analysis						Adjusted for *1 and folic acid supplement use						Multiple regression analysis					
							Multiple regression analysis						Adjusted for *2 and folic acid supplement use					
	R^2^	B	95%CI		β	p	R^2^	B	95%CI		β	p	R^2^	B	95%CI		β	p
Overall																		
Cognitive-adaptive DQ																		
0 ≤ – < 200	0.001	ref					0.060	ref					0.057	ref				
200 ≤ – < 400		0.809	− 0.273	1.891	0.028	0.14		0.866	− 0.409	2.140	0.030	0.18		0.751	− 0.369	1.870	0.026	0.19
≥ 400		0.174	− 1.313	1.661	0.004	0.82		1.263	− 1.207	3.734	0.032	0.32		0.990	− 0.845	2.824	0.025	0.29
Language-social DQ																		
0 ≤ – < 200	0.000	ref					0.089	ref					0.087	ref				
200 ≤ – < 400		0.636	− 0.565	1.837	0.020	0.30		0.807	− 0.586	2.200	0.025	0.26		0.705	− 0.528	1.939	0.022	0.26
≥ 400		0.239	− 1.412	1.890	0.006	0.78		1.593	− 1.108	4.293	0.037	0.25		1.402	− 0.560	3.364	0.032	0.16
Male offspring																		
Cognitive-adaptive DQ																		
0 ≤ – < 200	0.003	ref					0.063	ref					0.051	ref				
200 ≤ – < 400		1.353	− 0.249	2.955	0.045	0.10		1.303	− 0.606	3.211	0.044	0.18		1.020	− 0.642	2.682	0.034	0.23
≥ 400		− 0.864	− 3.085	1.357	− 0.021	0.45		0.715	− 2.958	4.387	0.017	0.70		− 0.281	− 2.833	2.270	− 0.007	0.83
Language-social DQ																		
0 ≤ – < 200	0.002	ref					0.079	ref					0.067	ref				
200 ≤ – < 400		1.094	− 0.682	2.870	0.033	0.23		1.362	− 0.734	3.457	0.041	0.20		1.147	− 0.677	2.971	0.035	0.22
≥ 400		− 0.432	− 2.894	2.030	− 0.009	0.73		1.847	− 2.185	5.880	0.040	0.37		0.924	− 1.890	3.737	0.020	0.52
Female offspring																		
Cognitive-adaptive DQ																		
0 ≤–< 200	0.001	ref					0.066	ref					0.056	ref				
200 ≤ – < 400		0.336	− 1.109	1.782	0.012	0.65		0.519	− 1.231	2.269	0.019	0.56		0.103	− 1.317	1.524	0.004	0.89
≥ 400		1.128	− 0.842	3.099	0.031	0.26		2.161	− 1.302	5.624	0.059	0.22		0.642	− 1.304	2.587	0.017	0.52
Language-social DQ																		
0 ≤ – < 200	0.000	ref					0.113	ref					0.105	ref				
200 ≤ – < 400		0.248	− 1.365	1.862	0.008	0.76		0.369	− 1.534	2.273	0.012	0.70		− 0.063	− 1.612	1.485	− 0.002	0.94
≥ 400		0.848	− 1.351	3.047	0.021	0.45		1.781	− 1.986	5.548	0.043	0.35		0.430	− 1.693	2.553	0.010	0.69

*DQ* developmental quotient, *B* partial regression coefficient, *CI* confidence interval, *Β(beta)* standardized partial regression coefficients, *R2* coefficient of determination.

*1: Adjusted for maternal age at delivery, maternal body mass index (kg/m2) before pregnancy, infertility treatment, unexpected pregnancies, parity, marital status, maternal highest level of education, paternal highest level of education, maternal smoking status during pregnancy, and paternal smoking status during pregnancy, maternal alcohol consumption during pregnancy, annual household income during pregnancy, pregnancy complications, obstetric labor complications, mode of delivery, maternal neuropsychiatric disorders, maternal Kessler 6 (K6) psychological distress scale scores >  = 5 during pregnancy, offspring’s sex (for overall) and birth weight, gestation week of delivery, breastfeeding at age of 18 months, family structure, maternal job after delivery, day care center attendance, multivitamin supplement use, iron preparations, trace element use, and the dietary intake (FFQ) included energy content and nutrients, including amino acids, n-3 unsaturated fatty acids, iron, calcium, vitamin A, vitamin B12, vitamin C.

*2: Variable selection was performed using a stepwise method. Adjusted for "Folic acid supplements use."

*2: Cognitive-adaptive DQ of Overall; Adjusted for maternal body mass index (kg/m2) before pregnancy, unexpected pregnancies, parity, marital status, maternal highest level of education, paternal highest level of education, paternal smoking status during pregnancy, maternal neuropsychiatric disorders, maternal Kessler 6 (K6) psychological distress scale scores >  = 5 during pregnancy, offspring’s sex (for overall) and birth weight, family structure, maternal job after delivery, day care centre attendance, iron preparations, trace element use, and the dietary intake (FFQ) included vitamin A.

*2: Language-social DQ of Overall; Adjusted for maternal age at delivery, maternal body mass index (kg/m2) before pregnancy, unexpected pregnancies, parity, maternal highest level of education, paternal highest level of education, paternal smoking status during pregnancy, annual household income during pregnancy, pregnancy complications, maternal Kessler 6 (K6) psychological distress scale scores >  = 5 during pregnancy, offspring’s sex (for overall) and birth weight, maternal job after delivery, day care centre attendance, iron preparations, and the dietary intake (FFQ) included calcium, vitamin B12.

*2: Cognitive-adaptive DQ of Male offspring; Adjusted for unexpected pregnancies, parity, marital status, maternal highest level of education, paternal highest level of education, paternal smoking status during pregnancy, offspring’s birth weight, day care center attendance, trace element use, and the dietary intake (FFQ) included vitamin B12.

*2: Language-social DQ of Male offspring; Adjusted for maternal age at delivery, maternal body mass index (kg/m2) before pregnancy, parity, maternal highest level of education, paternal highest level of education, paternal smoking status during pregnancy, maternal Kessler 6 (K6) psychological distress scale scores >  = 5 during pregnancy, offspring’s birth weight, iron preparations, and the dietary intake (FFQ) included vitamin B12.

*2: Cognitive-adaptive DQ of Female offspring; Adjusted for maternal highest level of education, paternal highest level of education, paternal smoking status during pregnancy, mode of delivery, maternal neuropsychiatric disorders, maternal Kessler 6 (K6) psychological distress scale scores >  = 5 during pregnancy, offspring’s birth weight, gestation week of delivery, family structure, day care centre attendance.

*2: Language-social DQ of Female offspring; Adjusted for maternal age at delivery, unexpected pregnancies, parity, maternal highest level of education, paternal highest level of education, pregnancy complications, maternal neuropsychiatric disorders, family structure, maternal job after delivery, day care centre attendance, iron preparations.

In male and female offspring, the multiple regression analysis without the stepwise method revealed no significant associations with any DQ score in the 200 µg to < 400 µg group and the ≥ 400 µg group compared with the < 200 µg group (Table 5). The multiple regression analysis with the stepwise method revealed no significant associations with any DQ score in the 200 µg to < 400 µg group and the ≥ 400 µg group compared with the < 200 µg group (Table 5).

## Discussion

Our study demonstrated that the offspring of mothers who started prenatal folic acid supplement use within 12 weeks of gestation exhibited better nonverbal and verbal cognitive development at age 4 than did those with mothers who did not use folic acid supplements. However, offspring of mothers with an adequate daily dietary folate intake from preconception to early pregnancy did not exhibit better nonverbal or verbal cognitive development at age 4 than did offspring of mothers with inadequate folate intake in that period. These results are inconsistent with those of our previous study on 2-year-old offspring ^8^.

First, our previous study of 2-year-old offspring ^8^ demonstrated that the offspring of mothers who took ≥ 200 µg folate per day from preconception to early pregnancy had a significantly higher DQ in the language-social area than did those in the < 200 µg group. Moreover, the DQ was higher in the ≥ 400 µg group than in the 200 to < 400 µg group. However, this study revealed that the beneficial association was no longer present in 4-year-olds. This suggests that the benefit of maternal dietary folate intake during early pregnancy on the offspring’s verbal cognitive development may last for up to approximately 2 years, and that postnatal environment factors may offset the difference between groups by the time the offspring are 4 years of age.

Second, regarding maternal prenatal folic acid supplementation, previous studies ^8^ and the current study were limited by the lack of detail on the amount of folic acid in the supplements used and the frequency of use. Our previous study of 2-year-old offspring revealed no significant association between starting prenatal folic acid supplement use within 12 weeks of gestation and verbal or nonverbal cognitive development. However, to our surprise, such associations were observed for 4-year-old offspring in this study. In Japan, only approximately 30% of pregnant women seem to start using folic acid supplements before conception or within 12 weeks of gestation ^14^. Therefore, pregnant women who use folic acid supplements may be more conscious of their future offspring’s health than women who do not ^21–23^. We hypothesize that mothers who use folic acid supplements in early pregnancy for their offspring’s health will exhibit enthusiastic parenting behavior after delivery. The effects of enthusiastic postpartum parenting behavior may become apparent when the offspring reach the age of 4. In support of this hypothesis, a cohort study in the Netherlands ^24^ revealed that there was no association between plasma folate concentrations in pregnant women and autistic traits in their offspring, but that prenatal folic acid use was associated with fewer autistic traits in the offspring at age 3. They suggested that prenatal folic acid supplement use, a marker of good health literacy, is associated with many health-conscious behaviors that decrease the background risk of autistic traits in offspring. To substantiate our hypothesis, the next task would be to analyze the relationship between maternal prenatal folic acid supplement use and postpartum parenting behavior.

In this study of 4-year-old offspring, we also explored sex differences in the effect of folic acid supplementation/dietary folate intake. Male offspring of mothers who started using folic acid supplements before conception or within 12 weeks of gestation exhibited better verbal cognitive development than those of mothers who did not use such supplements. However, no significant association was observed with nonverbal cognitive development. Female offspring of mothers who started using folic acid supplements within 12 weeks of gestation exhibited better nonverbal cognitive development than those of mothers who did not use such supplements. However, no significant association was observed with verbal cognitive development. In animal studies, sex differences have been demonstrated in maternal folic acid loading and behavior in offspring ^25,26^; however, few reports on such sex differences have been made for human studies. The reason for the observed sex difference in human offspring is unknown, and further investigation is required.

For reference, we compared our results to those of previous cohort studies on prenatal folic acid/dietary folate intake and cognitive development in offspring from 3 to 6 years of age^3^. Maternal supplement use of more than 600 µg/day and folate intake from food in early pregnancy were positively associated with receptive language development in 3-year-old offspring in a US cohort study ^27^. Maternal folic acid supplement use from the eighth week of pregnancy was associated with a reduced risk of severe language delay in 3-year-old offspring in a Norwegian cohort study ^28^. In a US cohort study, the use of periconceptional folic acid supplements was not associated with language development in 3-year-old offspring ^29^. In a Spanish cohort study, a positive association between maternal use of folic acid supplements at the end of the first trimester and social competence, verbal skills, and verbal-executive skills was observed in 4-year-old offspring. However, no differences in perceptive performance or memory were observed in that study ^30^. In a European, multicenter, randomized controlled trial, the maternal use of 400 μg/day folic acid supplement from the 20th week of pregnancy until delivery had no significant effect on the cognitive function of 6.5-year-old offspring ^31^. Moreover, a study in the US revealed that the folate nutritional status of mothers in the latter half of pregnancy, assessed via plasma and erythrocyte folate concentrations, had no impact on the cognitive development of 5-year-old offspring ^32^.

This section discusses the overall importance of folate/folic acid in pregnancy. Because the fetus receives folate from the mother through the placenta, pregnant women’s folate/folic acid intake must be adequate. Folate deficiency in pregnant women can cause megaloblastic anemia ^1^. Low folate status in pregnant women increases the risk of preterm delivery, low birth weight, fetal growth retardation, congenital heart disease, and structural malformations such as oral clefts ^1^. It has also been suggested that maternal folate deficiency may result in neurodevelopmental disorders such as autism spectrum disorders and schizophrenia in their offspring ^1^. It is also well known that supplementation with folic acid, a synthetic form of folate, reduces the prevalence of folate deficiency during pregnancy and that folic acid supplementation during gestation reduces the risk of neural tube defects (NTD) in the fetus^3–5^.

Besides folate/folic acid, other nutrients, such as protein, zinc, iron, vitamins, and long-chain polyunsaturated fatty acid, are also important for offspring’s neurodevelopment ^33–35^. The nurturing environment after birth is also important. Therefore, factors other than those we have included as confounding factors in this study may also likely play a role in children’s neurodevelopment. Further comprehensive evaluations that include folate/folic acid and these factors are needed.

This study had some limitations. The first limitation was the retrospective collection of information for maternal supplement use, which in the case of the preconception period was at least 10–16 weeks before the interviews; this may not have been very accurate. Second was the lack of detailed information on the use of folic acid supplements and whether the supplements used by all of the study participants contained the same amount of folic acid. In Japan, folic acid supplements are manufactured by various companies, but pregnant women and women planning to conceive are recommended to supplement their diet with 400 μg/day of folic acid, not exceeding 1000 μg/day ^12^. Thus, although not all pregnant women necessarily received the same dose of folic acid, each likely consumed at least 400 μg/day.Third, there was no accurate information on how long women took folic acid supplements during preconception or pregnancy. Fourth, the fact that dietary folate intake was self-reported via the FFQ. Fifth, there was no information on reliable biochemical indicators of folate status, such as red blood cell folate concentration, in the JECS study.

However, the study’s strength was in the objective investigation of the offspring’s cognitive development by trained interviewers.

In conclusion, our study demonstrated that maternal prenatal folic acid supplement use starting within 12 weeks of gestation was associated with higher verbal and nonverbal cognitive development in 4-year-old offspring than not using such supplements. However, there were sex differences in this association. Offspring of mothers with an adequate daily dietary folate intake from preconception to early pregnancy were not at an advantage in terms of verbal and nonverbal cognitive development at 4 years of age.

## Data Availability

Data are unsuitable for public deposition due to ethical restrictions and legal framework of Japan. It is prohibited by the Act on the Protection of Personal Information (Act No. 57 of 30 May 2003, amendment on 9 September 2015) to publicly deposit the data containing personal information. Ethical Guidelines for Epidemiological Research enforced by the Japan Ministry of Education, Culture, Sports, Science and Technology and the Ministry of Health, Labour and Welfare also restricts the open sharing of the epidemiologic data. All inquiries about access to data should be sent to: jecs-en@nies.go.jp. Te person responsible for handling enquiries sent to this e-mail address is Dr. Shoji F. Nakayama, JECS Programme Office, National Institute for Environmental Studies.
